# Egg Quality Parameters, Production Performance and Immunity of Laying Hens Supplemented with Plant Extracts

**DOI:** 10.3390/ani11040975

**Published:** 2021-03-31

**Authors:** Muhammad Ammar Dilawar, Hong Seok Mun, Dhanushka Rathnayake, Eun Ju Yang, Ye Seul Seo, Hyeoung Seog Park, Chul Ju Yang

**Affiliations:** 1 Interdisciplinary Program in IT-Bio Convergence System (BK 21 Plus), Sunchon National University, 255, Jungang-ro, Suncheon-si, Jeollanam-do 57922, Korea; ammar_dilawar@yahoo.com; 2 Animal Nutrition and Feed Science Laboratory, Department of Animal Science and Technology, Sunchon National University, 255 Jungang-ro, Suncheon, Jeollanam-do 57922, Korea; mhs88828@nate.com (H.S.M.); dhanus871@gmail.com (D.R.); 3 Food Research Center, Jeonnam Bio Industry Foundation, Naju 58275, Korea; rootage@hanmail.net (E.J.Y.); yesual0314@naver.com (Y.S.S.); 4 EFC, Gwangyang 57714, Korea; hsparkblue@hanmail.net

**Keywords:** egg quality, egg production, yolk cholesterol, laying hens

## Abstract

**Simple Summary:**

The current study aimed to investigate the effects of a combination of two plant extracts *Mentha arvensis* and *Geranium thunbergii* in drinking water. A complete randomized design was performed to study the effects on production performance, egg quality parameters, sensory qualities, proximate analysis, and cholesterol content of egg yolk and immunity parameters in laying hens. The data suggested that the supplementation of plant extracts could improve the laying performance, egg quality and immunity, and decrease the egg yolk cholesterol content in a dose-dependent manner.

**Abstract:**

This study examined the effects of *Mentha arvensis* (MA) and *Geranium thunbergii* (GT) extracts in drinking water on the production performance, egg quality, cholesterol content of egg yolk, proximate composition, and sensory qualities of egg and immunity parameters in laying hens. Ninety-six 28-week-old Hy-Line Brown layers were randomly divided into four dietary treatments for 16 weeks. The dietary treatments were (1) control, (2) T1 (0.01% 1 MA:1 GT), (3) T2 (0.05% 1 MA:1 GT), and (4) T3 (0.1% 1 MA:1 GT). Egg production increased significantly with increasing levels of MA and GT. The egg weight was increased in T2, and the feed intake was highest in T2 and T3 (*p* < 0.05). The Haugh unit and egg shape index were significantly better in T3 and the control than with other treatments (*p* < 0.05). The content of yolk cholesterol was significantly lower (*p* < 0.05) in T2 and T3. On the other hand, there were no significant differences in the egg proximate composition. A significant increase in the serum interleukin 6 (IL-6), tumor necrosis factor (TNFα) and immunoglobulins (IgG and IgA) concentration was observed in the birds fed plant extracts when compared to the control. On average, T2 and T3 showed significantly lower (*p* < 0.05) concentrations of NH_3_ gas from the feces as compared to the control. This study suggests that MA and GT supplementation could improve the laying performance, egg quality, and immunity, and decrease the egg yolk cholesterol content in a dose-dependent manner.

## 1. Introduction

Eggs are considered to be a “miracle food”, because they contain approximately 40 proteins, including antihypertensive and bactericidal proteins, 18 different amino acids, including nine essential amino acids, stable amino acid composition, optimal proportion of saturated and unsaturated fatty acids, and no carbohydrates or trans fats. Therefore, eggs have been recognized as a reference protein for humans and they have the same biological value as breast milk [1,2]. The global production of table eggs has increased by 24.4% over the past decade, bringing production to 76.7 million tonnes in 2018, which is expected to increase further because of the high demand for animal-originated protein [3]. This high demand has triggered the need for intensive poultry production, which causes an increased incidence of disease, chronic stress, and compromised production [4]. Over the past decades, antibiotics have been regularly included in layer diets to enhance the performance and prevent diseases, with the ultimate production of safe and good quality eggs [5]. On the other hand, the poultry industry faces a ban on the sub-therapeutic use of antibiotics as a growth promoter because of the development of drug-resistant bacteria in humans [6]. This had led to searches for bioactive compounds that could serve as effective substitutes for antibiotics. The bioactive compounds obtained from plants have been employed in poultry diets to enhance the production performance and immune status [7]. The beneficial effects of phytobiotics as individual compounds or mixed preparations in poultry include the improvements in the production performance parameters, efficient utilization of nutrients, boosting the immune system, and antioxidant and antibacterial properties [8].

*Mentha arvensis* (MA) is a plant species in the Lamiaceae family that is widely used in herbal medicine. The bioactive compounds of this plant include menthol, isomenthol, neomenthol, p-cymene, d-menthone, eugenol, and cineol [9,10], which are responsible for its phytochemical properties, including increased appetite, activating the immune system, and antibacterial and antioxidant properties [11]. *Geranium thunbergii* (GT) is a perennial plant species belonging to the Geraniaceae family and it is found in China, Korea, and Japan. The main bioactive components of this plant are citronellol, isomenthone, and geraniin that possess anti-inflammatory, antibacterial, antioxidant, antihypertensive, and antifungal effects [12].

Despite the beneficial effects of both plants, there is a paucity of research on their use in animal diets. Previous studies evaluated the effects of both plant extracts on broiler and pig production performance and meat quality [13,14,15]. Therefore, this study was conducted to investigate whether the inclusion of MA and GT in drinking water would improve egg production, egg quality, cholesterol content of eggs, and sensory and immunity parameters in laying hens.

## 2. Materials and Methods

### 2.1. Animal Care

The Animal Care and Use Committee (SCNU IACUC 2019-05), Sunchon National University, Korea approved the study protocols.

### 2.2. Birds, Housing, Diet and Management

Ninety-six ISA Brown laying hens, 28-week of age, were randomly assigned to four treatment groups with six replicate cages having four hens per cage. The hens were kept at a stocking density of 700 cm^2^/ hen, a photoperiod of 16 h/8 h light/dark cycle, and a room temperature of 20 °C ± 3 °C. The experiment lasted for 16 weeks, and the birds were given access to feed and water *ad libitum* (with the inclusion of dietary treatments) throughout the experimental period. Commercially available laying hen feed (Nonghyup feed, Gyeongsangnam-do, Korea) was fed a basal diet. Table 1 list the ingredients and calculated the chemical composition of the diet. The extracts of two experimental plants (i.e., MA and GT) were prepared using the method that was described by Dilawar et al. [13]. Briefly, the MA and GT extracts were prepared by using 100 g of leaves and 5 L distilled water. The mixture was kept at room temperature for 2 h with occasional shaking. The same method was repeated for the extraction of 2 kg dried leaves, after which the samples were filtered with the help of Whatman No. 1 filter paper. The combination treatments were prepared in the broiler house before adding to the drinking water. The dietary treatments were: (1) control (0% MA:GT), (2) T1 (0.01% 1 MA:1 GT), (3) T2 (0.05% 1 MA:1GT), and (4) T3 (0.1% 1 MA:1 GT).

### 2.3. Performance Parameters

The eggs were collected manually at 08.00 a.m. each day. The weight and number of eggs laid were recorded daily. The egg weight was determined by a HM-200 electronic scale (A&D Co., Ltd., Tokyo, Japan). The abnormal eggs (broken eggs, shell-less, or soft shells) were excluded when measuring the egg weight. The egg production percentage was calculated as the hen-day egg production (HDEP) and egg mass were calculated by multiplying the average egg weight with HDEP. The percentage of broken eggs was calculated by dividing the number of broken and soft-shell eggs by the total number of eggs laid.

Fresh feed and water (with the inclusion of treatments) were offered daily on an *ad libitum* basis. The feed and water intake were determined weekly by measuring the residues. The feed conversion ratio (FCR) was calculated by dividing the feed intake by the egg mass).

### 2.4. Egg Quality Parameters

The egg quality indices were determined every week by randomly collecting 12 eggs per treatment. The eggshell thickness was measured using a Peacock dial gauge (P-1 Model, Meg Co Ltd., Ozaki, Japan) after removing the shell membrane and it is represented as the average thickness of the upper, middle, and lower end of the shell. The Haugh units (HU), albumin height, and yolk color were analyzed using an Egg Multi Tester EMT-5200 (Robotmation Co. Ltd., Tokyo, Japan). The egg length (L) and width (W) were measured using Vernier calipers with the least count of 0.01 mm. The egg shape index (SI) was determined from the egg length and width that were described by Duman et al. [16].

### 2.5. Egg Yolk Cholesterol Content

The cholesterol content in egg yolk was determined by collecting 48 eggs (12 eggs per treatment) in the last week of the experiment. Briefly, 1 g egg yolk sample and 100 µg of 5α-cholestane were homogenized with 22 mL ethanol and 0.5 N KOH (aqueous) and then saponified at room temperature at 6 h. The total cholesterol content in the egg yolk was extracted with hexane and then analyzed by gas chromatography (Agilent, 7890B series, CA, USA) equipped with an ionization detector and a HP-5 (J&W, 30 m × 0.25 mm × 0.25 µm) capillary column. The chromatographic parameters were as follows: sample injection volume 1 µL, split ratio 1:50, and the detector and injector temperatures were both 280 °C. Nitrogen gas (400 mL/min.) was used as the carrier gas, and hydrogen gas (40 mL/min.) was used as a makeup gas. The cholesterol content is expressed as mg/100g egg yolk.

### 2.6. Proximate Composition

The proximate composition of the eggs was analyzed by collecting 48 eggs (12 eggs per treatment) in the last week of the experiment. The eggs were broken manually and then homogenized with a homogenizer (Ultra-Turrax GMBH & Co. IKA Werke, Staufen, Germany). The moisture (method 925.30 using a dry oven), crude ash (method 900.02 using a muffle furnace), crude protein (method 925.31 using KjelMaster K-375, BÜCHI Labortechnik AG., Flawil, Switzerland), and crude fat or ether extract (method 925.32 using the Soxtec 2500, LabMakelaar Benelux B.V., Zevenhuizen, The Netherlands contents of the egg were determined according to the Association of Official Analytical Chemists AOAC [17].

### 2.7. Sensory Evaluation of Eggs

Twelve trained judges (six male members and six female members aged 20–40 years) from Sunchon National University, Korea, were selected for the sensory evaluation of the eggs. The sensory evaluation was performed using a slight modification of the method that was reported by Hayat et al. [18]. Six eggs from each treatment were added to a 1.5-quart (approx. 1450 mL) stainless steel pot with a lid, which contained 48 oz. (approx. 1350 mL) of tap water. The gas burner was turned on and kept at high heat level for 8.5 min. until the eggs were brought to a low-rolling boil. After turning off the heat, the eggs were kept in the pot with a lid for 20 min. The water was drained, and the eggs were kept under running tap water until the temperature of the egg was cooled to room temperature. The eggs were peeled and cut into quarters (lengthwise) for the sensory evaluation. The sensory attributes tested were, as follows: (a) odor, aroma/smell of the whole boiled egg; (b) off-flavors, unpleasant or unusual taste or smell of the boiled egg; (c) flavor, the distinctive taste and aroma of the boiled egg; (d) color, overall color of the whole boiled egg; and (e) palatability, an integrated palatable sensation based on the above parameters. The panelists were asked to rate the eggs on a nine-point intensity scale where 0 = extremely bad, 5 = moderately acceptable, and 9 = highly acceptable. The judges were also provided with cold water in between the samples to rinse their mouth.

### 2.8. Immunoglobulin and Cytokines Detection

At the end of the experiment, three birds per replication were randomly selected for the detection of immunoglobulins (IgG and IgA) and cytokines, i.e., interleukin 6 (IL-6) and tumor necrosis factor (TNFα). The blood samples (5 mL) were collected from the brachial wing vein into a 10 mL anticoagulant-free vacutainer (Greiner Bio-One GmbH, Kremsmunster, Austria). The serum was immediately separated by centrifugation for 15 min. at 1610× *g* at 4 °C and then stored in plastic vials at −20 °C until further analysis.

The concentrations of IgA, IgM, and IgG in serum were analyzed using sandwich chicken ELISA kits (Bethyl Laboratories Inc., Montgomery, TX, USA) with chicken-specific IgA (Cat. No. E30-103) and IgG (Cat. No. E30-1-4). A microplate auto reader (Thermo Lab Systems, Finland) was used to measure the absorbance of each well at 450 nm within 30 min. The concentrations of immunoglobulins were measured using standard curves that were obtained from standard immunoglobulin and then expressed as mg/mL of serum.

An analysis of the IL-6 and TNFα concentration in serum was detected using chicken IL-6 and TNF-α ELISA kits (NovaTein Biosciences, MA, USA). Briefly, 50 µL of serum and 100 µL of a conjugate solution were added to a micro-ELISA strip plate well and allowed to react for 1 h. After washing, 50 µL of each of chromogen solution A and B were added to the well and then allowed to react for 15 min. at 37 °C, then stopped by adding 50 µL of a stop solution. The absorbance was measured at 450 nm, and the concentration of cytokines was measured using standard curves and expressed as pg/mL of serum.

### 2.9. Odorous Gas Emission and pH of Excreta

The concentration of ammonia (NH_3_) was measured by collecting 300 g of excreta sample from the bottom tray of each replicate cage in a plastic zipper bag. The bags were placed in a plastic box with a lid having two holes. One hole was sealed with a membrane filter of pore size 1.0 µm (Advantec^®^, Toyo Roshi Kaisha Ltd., Tokyo, Japan), and another was used to measure the gas emission. The samples were allowed to ferment at room temperature, and gas emission was recorded using a Gastec gas-sampling pump (AP-20, Gastec Corp., Kitagawa, Japan) and a detector tube (3 LA, 3M for NH_3_) at 0, 6, 12, 24, and 48 h. The NH_3_ concentration was expressed as ppm/100 mL.

The pH of feces was measured using a digital pH meter (Docu-pH meter, Sartorius, USA) after diluting 1 g of a fecal sample with 9 mL of distilled water.

### 2.10. Statistical Analysis

The data were analyzed by one-way analysis of variance (ANOVA) using the General Linear Model function (GLM) of the SAS Statistical Package Program (SAS, 2005, Version 9.0, Cary, NC, USA). The following statistical model was used to determine the effects of the treatment (Equation (1)):

Y_ij_ = µ + α_ij_ + e_ij._ (1)

where µ = general mean, e_ij_ = random error, α_i_ = effect of dietary treatments, and Y_ij_ = response variable. The means were calculated and presented with the standard error of the mean (SEM). The differences among treatments were analyzed by Duncan’s multiple range test, and the results were considered to be significant if the *p*-values were equal to or less than 0.05.

## 3. Results and Discussion

### 3.1. Production Performance

The egg weight, HDEP, egg mass, broken egg rate, feed intake, FCR, and water consumption were evaluated as an indicator of layer production performance (Table 2). The egg weight was significantly increased in T2 than in the control group (*p* < 0.05). The T2 and T3 group had a significantly higher HDEP, egg mass, and feed intake relative to the control. On the other hand, the broken egg rate and FCR were not influenced by the dietary treatments. The diet that was supplemented with the T3 treatment reduced water consumption as compared to that of the control (*p* < 0.05).

The prohibition against antibiotics as growth promotors has prompted significant research into phytochemicals as alternatives. The beneficial effects of dietary supplementation of plant extracts on gut health, intestinal integrity, and better utilization of nutrients have been reported [19,20], which can be linked with the improvements in the laying hen performance. In the present study, the birds that were supplemented with MA and GT had the highest egg production, egg mass, and egg weight. These results agree with Abdel-Wareth and Lohakare [19], who reported that feeding peppermint to laying birds had a positive influence on the conversion of digested feed into eggs, which is crucial for the oviposition process. Moreover, the bioactive components in both tested herbal plants have antimicrobial and antioxidant properties [21,22], and they play a vital role in the digestion and absorption of nutrients [23] that might have improved the performance parameters of laying hens. An increased feed intake was observed in T2 and T3, which can be explained by the core mode of the nutritional action of phytogenic feed additives including the stimulation of digestive enzymes and enhanced digestive secretions [24]. Consistent with these findings, an increased feed intake was also reported earlier in broilers, and layers fed MA and GT extracts [13,19,22]. On the other hand, the non-significant but better FCR observed due to the inclusion of plant extracts could be due to the increased feed utilization efficiency [19]. Furthermore, a lower water consumption was observed in T3 due to the inclusion of plant extracts in the drinking water at a higher level. This may be due to dose-related depression of palatability [23], or the characteristic smell of the plant extracts had transferred to the drinking water of birds. Therefore, it could be stated that the extracts of MA and GT have a positive response on production performance parameters of laying hens attributed to the presence of bioactive compounds.

### 3.2. Egg Quality Parameters

Table 3 lists the effects of MA and GT on the egg quality parameters. The egg shape index was significantly increased (*p* < 0.05) in T3 relative to the control. The dietary supplementation did not affect the egg height, yolk height, and eggshell thickness. The Haugh unit was significantly better in T3 when compared with the control and other treatments (*p* < 0.05).

Egg quality refers to various standards that define both external and internal quality. The internal quality is focused on the yolk height, yolk color, albumin viscosity, and Haugh unit. In contrast, the external quality refers to the eggshell thickness, egg width, and height and cleanliness [25]. The Haugh unit is a measure of egg freshness and it is related to the shelf life [26]. The eggshell thickness is associated with the proportion of damaged eggs during transport and handling [27]. This study observed no significant differences in yolk color, egg width, egg height, and eggshell thickness. Previously, Abdel-Wareth and Lohakare [19] reported that peppermint oil increased the eggshell thickness, which was not found in the present study. On the other hand, there was a significant effect on the Haugh unit and egg shape index (SI) in T3 due to the supplementation of two plant extracts. These findings are in agreement with earlier studies that the herbs included in the Mentha genus improved the Haugh unit in laying hens [19,28]. Unfortunately, few studies have been conducted. Of those, there have been contradictory results on the effect of phytobiotics and their mode of action on the egg quality in laying eggs [29]. Therefore, further research will be needed to explore the effect of herbal plants on the egg quality parameters, particularly in regard to their mode of action, as the results vary significantly by the difference in quality and proportion of active components that are present in each plant extract [24].

### 3.3. Egg Yolk Cholesterol and Proximate Composition

The proximate composition of eggs was similar, regardless of the dietary treatments (Table 4). On the other hand, the egg yolk cholesterol content was significantly lower in the T2 and T3 supplemented groups than in the control group (*p* < 0.05).

To the best of the authors’ knowledge, there are little data available regarding the effects of the MA and GT extracts on the proximate composition and egg yolk cholesterol content in commercial laying hens. The nutritional value and proximate composition of animal-derived products depend on the diet, genetics, age, management, and feeding of the source animal [14]. In the present study, the proximate egg composition, such as moisture, crude protein, fat, and ash, did not differ between the dietary treatments. A non-significant decrease in crude fat content in eggs was observed because of the addition of MA and GT in drinking water. In contrast, Devi et al. [30] reported that the protein content in eggs was increased due to MA supplementation, which might be due to the high level (10 g/kg) of tested plants that were used in their study.

The dietary treatments (T2 and T3) in this study reduced the cholesterol levels in egg yolk. In contrast, the low level of plant extracts in T1 have negative effect on the cholesterol level, which needs to be further evaluated. Sometimes, the decrease in egg cholesterol content had a negative effect on egg production [31], which was not observed in the present study. The cholesterol-lowering effects of MA and GT in farm and experimental animals were reported. For example, the dietary supplementation of peppermint and GT lowered cholesterol levels in laying hens and mice, respectively [12,19]. Cholesterol in egg yolk is synthesized in the liver, secreted as very-low-density lipoprotein (LPL) in blood, and deposited via receptor-mediated endocytosis in yolk [32]. Thus, the decrease in cholesterol by MA and GT supplementation might be due to the consequences of altered endogenous lipoproteins (production or secretion) or/and cholesterol metabolism (i.e., synthesis, degradation, and distribution) [31]. Furthermore, Akbarian et al. [33] reported that the phenolic compounds in plant extracts have an inhibitory effect on 3-hydroxy-3methylglutaryl coenzyme A, which is essential for cholesterol synthesis. These findings suggest that MA and GT have potential as feed additives in the production of low-cholesterol eggs, which consumers would prefer because cholesterol is a risk marker for coronary heart diseases and stroke [34].

### 3.4. Sensory Evaluation of Eggs

The supplementation of MA and GT in drinking water did not affect sensory parameters of eggs, except off-flavor (Table 5). The panelist found that eggs from the control were off-flavor (*p* < 0.05) as compared with dietary treatments.

The sensory or organoleptic properties of eggs, such as flavor, aroma, color, and palatability, are essential for customers [18]. In this study, the panelists did not identify any negative impact that is caused by supplementation of the two plant extracts. The sensory evaluation of eggs from laying hens fed MA and GT has not been reported before, which limits the possibility of comparing data. The eggs collected from the birds fed MA and GT have better palatability in T3 (5.71) than the control (5.25), but the results were not statistically significant. These data suggest that aromatic compounds and the characteristic smell of plant extracts are not transferred to the eggs. In terms of the off-flavor, the eggs obtained from the birds that were supplemented with MA and GT were better than the control. This may be due to the antioxidant potential of MA and GT [12,35], which prevents the oxidation of proteins and lipids in egg yolk, resulting in better flavor.

### 3.5. Immunity Parameters

**Figure 1:**
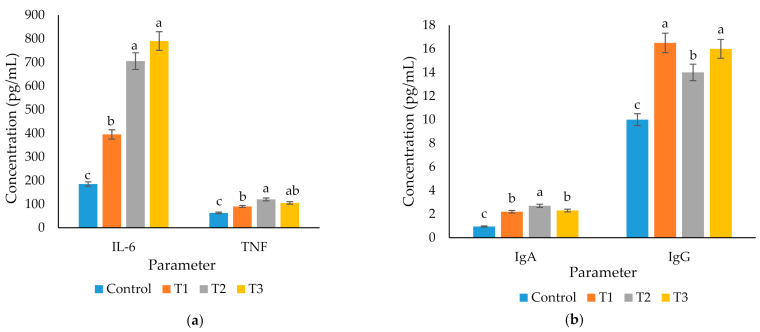
Effects of *Mentha arvensis* (MA) and *Geranium thunbergii* (GT) on immunity parameters. (**a**) Concentration of serum (IL-6) and tumor necrosis factor (TNFα); (**b**) Concentration of serum immunoglobulins IgA and IgG. ^a,b,c^ Bars within a particular point not sharing a common letter differ significantly (*p* < 0.05). *n* = Each value represents the mean of 6 replications with 3 birds per replicate.

A significant increase was observed in the serum IL-6 and TNFα concentration of birds fed plant extracts than the control (Figure 1a). Similarly, the concentration of IgG and IgA in the serum was increased (*p* < 0.05) due to the supplementation of MA and GT relative to the non-supplemented group (Figure 1b).

Secondary metabolites (flavonoids, flavanones, phenols, and saponins) in herbal plants have been suggested to possess immunomodulatory activities in animals [8]. The immuno-stimulatory properties of many phytogenics have been widely studied in poultry. For example, Ginseng has immuno-stimulatory properties, including macrophage activation and cytokine production [36]. Lillehoj et al. [37] reported the immuno-stimulatory effects of a 100% botanical officially approved phytonutrient formulation in Europe, CCC (cinnamaldehyde + carvacrol + capsicum). In the present study, the dietary supplementation of MA and GT improved the immune status of laying birds by increasing the concentration of serum IgA and IgG. Similarly, the production of IL-6 and TNFα was also higher in the supplemented groups. These results agree with previous findings that the flavonoids and saponins in various plant species enhance antibody production [38], macrophage action, and stimulate IL-6 and TNFα activity [36].

IL-6 and TNFα are the most investigated pro-inflammatory cytokines that are expressed by macrophages and monocytes after invading pathogens are recognized and initiate metabolic changes that boost the immune system and disease resistance [39]. Ghareeb et al. [40] reported that the expression of TNFα and IL-6 is strongly linked with the immune status of poultry. An improvement in the immune status of birds fed MA and GT is believed to be induced due to (i) the total effect of secondary metabolites present in both plant extracts [41], (ii) production of heat shock proteins that enhance immune surveillance of infected cells and boost immunity, and (iii) initiation of Toll-like receptors and immune activation [8]. Consistent with the present findings, various studies [42,43] have shown that the presence of phenolic compounds, flavonoids, and carvacrol in phytobiotics lead to a higher level of immunoglobulins and enhanced immune response in poultry. Despite the studies on the immuno-stimulatory effects of herbal plants, the precise mechanism of action of individual secondary metabolites is unknown. Hence, further research will be needed in order to understand the association between herbal plants and the immune system clearly.

### 3.6. Odorous Gas Emission and pH of Excreta

**Figure 2:**
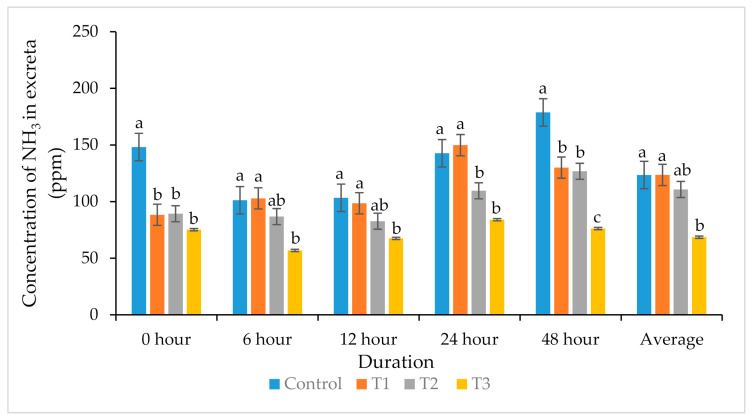
Effects of *Mentha arvensis* (MA) and *Geranium thunbergii* (GT) on concentration of NH_3_ emission from excreta. ^a,b,c^ Bars within a particular point not sharing a common letter differ significantly (*p* < 0.05). *n* = Each value represents the mean of six replications with four birds per replicate.

Figure 2 presents the emission of NH_3_ from the excreta. After 0, 6, 12, 24, and 48 h of storage, T3 decreased (*p* < 0.05) the concentration of NH_3_ gas from the feces relative to the control.

**Figure 3:**
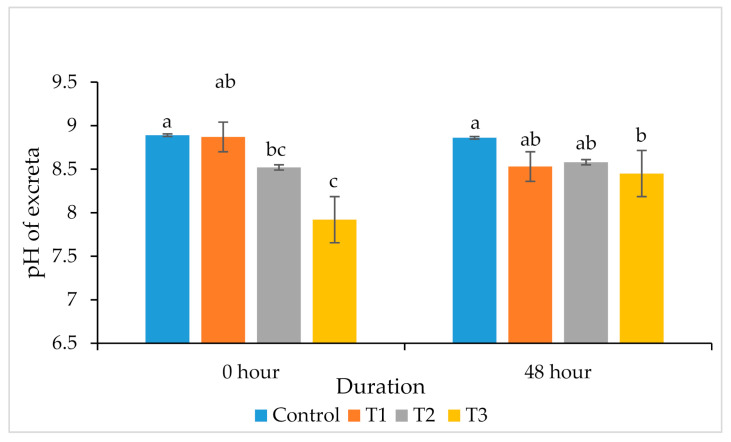
Effects of *Mentha arvensis* (MA) and *Geranium thunbergii* (GT) on the pH of excreta. ^a,b,c^ Bars within a particular point not sharing a common letter differ significantly (*p* < 0.05). *n* = Each value represents the mean of 6 replications with 4 birds per replicate.

Similarly, supplementation of MA and GT in T3 decreased the pH of the excreta significantly (*p* < 0.05) after 0 and 48 h when compared to the control group (Figure 3).

An important factor that determines the emission of NH_3_ from excreta of animals is the pH of manure [44]. A lower fecal pH limits the conversion of ammonia-nitrogen to NH_3_, which reduces the emission of NH_3_ into the atmosphere. NH_3_ emission from poultry farms is an alarming concern for environmental pollution, because it adversely affects the health of workers and birds, resulting in depressed performance [45]. Supplementation with tested plant extracts at the 0.1% level (T3) significantly reduced the pH of excreta at 0 and 48 h, ultimately resulting in a decrease in NH_3_ production. These results agree with a previous study [13], in that the supplementation of MA and GT reduced NH_3_ production (7–34.4%) from broiler excreta. Li et al. [46] suggested that the release of NH_3_ from poultry houses can be minimized by manipulating the diet without affecting the birds’ production and performance. The decreased pH is the main reason for the decrease in noxious gas, because the growth of ureolytic bacteria that facilitate NH_3_ production requires a high pH for growth [47]. Modification of the gut microbiota increased the digestion and better absorption of nutrients. Furthermore, the antimicrobial activities of plant extracts may also contribute towards the decrease in excreta pH and mitigating ammonia [48].

## 4. Conclusions

It is concluded that *Mentha arvensis* and *Geranium thunbergii* supplementation improved egg production and decreased the egg yolk cholesterol content. The dietary *Mentha arvensis* and *Geranium thunbergii* also increased the serum interleukin-6, tumor necrosis factor α, Immunoglobulins (IgA and IgG), and reduced the emission of harmful ammonia gas from the excreta.

## Figures and Tables

**Table 1:** Formula and chemical composition of layer diet.

Ingredients (%)	
Corn grain	45.50
Wheat	8.90
Dried distillers grains with solubles	7.00
Soybean meal (44% CP)	13.75
Canola meal	5.00
Corn gluten meal	4.00
Full-fat rice bran	3.50
Tallow	0.90
L-lysine HCl	0.18
DL-methionine	0.09
Dicalcium phosphate	1.20
Limestone	9.50
Salt	0.15
Sodium bicarbonate	0.10
Vitamin-mineral mixture ^1^	0.20
Phytase	0.03
Calculated composition (% DM)	
AME_n_ (kcal/kg) ^2^	2750
Crude protein (%)	17.00
Crude fat (%)	4.20
Lysine (%)	0.85
Methionine + cysteine (%)	0.70
Calcium (%)	4.10
Available Phosphorous	0.35

^1^ Vitamin-mineral mixture provided following nutrients per kg of diet: vitamin A, 9000 IU; vitamin D3, 3000 IU; vitamin E, 20 IU; Vitamin K3, 2 mg; vitamin B1, 1.5 mg; vitamin B2, 4.5 mg; vitamin B6, 3.0 mg; vitamin B12, 0.012 mg; Fe, 50 mg; Zn, 106 mg; Mn, 120 mg; Cu 11.2 mg; I, 1.7 mg; Se, 0.55 mg; Cr, 0.25 mg. ^2^ AMEn = nitrogen-corrected apparent Metabolizable energy.

**Table 2:** Effects of *Mentha arvensis* (MA) and *Geranium thunbergii* (GT) on egg performance of laying hens.

Item	Dietary Treatment (*n* = 24)				SEM	*p*-Value
	Control	T1	T2	T3		
Egg weight (g)	60.45 ^c^	61.33 ^bc^	62.06 ^a^	60.53 ^c^	2.63	0.0001
Hen-day egg production (%)	84.11 ^b^	86.46 ^ab^	89.49 ^a^	89.66 ^a^	5.66	0.0130
Egg mass (g/bird/d)	51.04 ^b^	51.90 ^ab^	54.91 ^a^	53.48 ^a^	9.81	0.0236
Broken egg rate (%)	1.13	1.02	1.14	0.95	2.79	0.4586
Feed intake (g)	114.68 ^bc^	110.34 ^c^	117.60 ^a^	116.79 ^a^	9.82	0.0444
FCR	2.25	2.13	2.14	2.18	0.43	0.3715
Water consumption (mL)	180.13 ^b^	183.69 ^ab^	187.44 ^a^	172.00 ^c^	5.94	0.0001

^a,b,c^ Values with different superscripts differ significantly (*p* < 0.05).

**Table 3:** Effects of *Mentha arvensis* (MA) and *Geranium thunbergii* (GT) on the egg quality parameters.

Item	Dietary Treatment (*n* = 12)				SEM	*p*-Value
	Control	T1	T2	T3		
Yolk height (mm)	6.36	5.88	5.92	6.30	2.11	0.0076
Yolk color	7.91 ^a^	7.53 ^bc^	7.41 ^c^	7.64 ^abc^	1.15	0.0234
Haugh Unit	71.07 ^bc^	66.65 ^c^	70.70 ^bc^	76.96 ^a^	6.98	0.0007
Egg height (cm)	5.33 ^ab^	5.39 ^a^	5.37 ^a^	5.30 ^b^	0.23	0.0409
Egg width (cm)	4.20	4.34	4.25	4.23	0.19	0.6203
Egg shell thickness (mm)	0.40 ^ab^	0.39 ^b^	0.41 ^a^	0.40 ^ab^	0.04	0.0357
Egg shape index	78.89 ^b^	78.73 ^b^	79.22 ^ab^	79.99 ^a^	3.02	0.0454

^a,b,c^ Values with different superscripts differ significantly (*p* < 0.05).

**Table 4:** Effects of *Mentha arvensis* (MA) and *Geranium thunbergii* (GT) on egg proximate composition (%) and cholesterol content (mg/100 g).

Item	Dietary Treatment (*n* = 12)				SEM	*p*-Value
	Control	T1	T2	T3		
Moisture	80.25	80.72	80.74	80.85	0.59	0.3482
Crude protein	14.56	14.22	14.55	14.42	0.30	0.3713
Crude fat	4.19	4.14	3.73	3.74	0.53	0.3961
Crude ash	1.01	0.92	0.97	0.99	0.07	0.1557
Cholesterol	178.70 ^b^	229.28 ^a^	144.89 ^c^	159.77 ^c^	0.94	0.0001

^a,b,c^ Values with different superscripts differ significantly (*p* < 0.05).

**Table 5:** Effects of *Mentha arvensis* (MA) and *Geranium thunbergii* (GT) on egg sensory evaluation.

Item	Dietary Treatment (*n* = 6)				SEM	*p*-Value
	Control	T1	T2	T3		
Odor	5.21	5.25	5.54	5.79	1.14	0.3397
Flavor	5.08	5.67	5.83	5.50	1.40	0.3124
Off-flavor	4.67 ^a^	3.88 ^ab^	3.58 ^b^	3.71 ^b^	1.38	0.0470
Palatability	5.25	5.63	5.63	5.71	1.37	0.7483
Color	5.33	5.50	5.25	5.46	1.31	0.2473

^a,b^ Values with different superscripts differ significantly (*p* < 0.05).

## Data Availability

Data is contained within this article.
